# Effect on the Quality of *Larimichthys crocea* Pretreated with Dual-Frequency Orthogonal Ultrasonic-Assisted Immersion with Different Powers during Refrigerated Storage at 4 °C

**DOI:** 10.3390/foods12173259

**Published:** 2023-08-30

**Authors:** Chenchen Zhang, Jinfeng Wang, Jing Xie

**Affiliations:** 1 College of Food Science & Technology, Shanghai Ocean University, Shanghai 201306, China; m210310953@st.shou.edu.cn (C.Z.); jfwang@shou.edu.cn (J.W.); 2 Shanghai Professional Technology Service Platform on Cold Chain Equipment Performance and Energy Saving Evaluation, Shanghai Ocean University, Shanghai 201306, China; 3 National Experimental Teaching Demonstration Center for Food Science and Engineering, Shanghai Ocean University, Shanghai 201306, China; 4 Shanghai Engineering Research Center of Aquatic Product Processing & Preservation, Shanghai Ocean University, Shanghai 201306, China

**Keywords:** large yellow croakers, ultrasound, pretreatment, orthogonal dual frequency, cold storage, quality characteristics

## Abstract

In recent years, ultrasonic pretreatment technology has been widely used in the aquatic product preservation industry. Among these technologies, dual-frequency ultrasonic refrigeration is the most common. However, in practical applications, selecting the frequency is relatively simple, and there has been less research on power selection. In this paper, the specific frequency (up and down 20 kHz, around 40 kHz), using different powers of (a) 200 W, (b) 300 W, and (c) 400 W processing, ultrasonic intermittent mode with 30 s on/30 s off cycle, and an ultrasonic processing time of 10 min was examined; the control group (CK) comprised samples without ultrasonic treatment. The samples were stored at 4 °C and then placed in a Polyethylene (PE) bag. The changes in microbiological parameters, physicochemical indices, and protein indices of the samples were monitored every two days. The results show that 400 W ultrasonic treatment can significantly inhibit the growth of TVC during storage. The rate of increase in pH, TVB−N, and TBA values decreased significantly compared with the other groups. Compared with the CK group, the shelf life of the 400 W treatment group was extended by 6 days. Therefore, the 400 W pretreatment method based on orthogonal double frequency has strong application potential for effectively extending the shelf life of refrigerated large yellow croaker.

## 1. Introduction

Under the genus Tototoya, a fish called the large yellow croaker (LYC) is also known as *Larimichthys crocea*. It is a common marine fish found in the East China Sea in China as well as the southern Yellow Sea and the Northwest Pacific that span the coastal areas of Vietnam, Korea, Japan, and China. Such croakers possess high levels of protein content and tenderness, and therefore have monetary value [1]. However, they only have a limited storage life after being butchered for consumption, which is primarily caused by the proliferation of bacteria. Hence, in the aquaculture industry and for consumers, it is crucial to maintain their highest possible quality while extending their shelf life [2].

An ultrasonic wave is a sound wave greater than 20 KHZ that has the advantages of long sound waves and high frequency. Additionally, it produces many special effects (e.g., chemical, thermal, and mechanical effects, among several others) and cavitation [3]. The improving effect of ultrasonic-assisted treatment on aquatic product quality is attributable to its “acoustic cavitation” effect, the essence of which is the rapid formation and collapse of tiny bubbles in a liquid medium due to waves of pressure [4]. The bactericidal effects of ultrasonic waves are fundamentally due to cavitation, as agreed upon by the majority of experts [5]. In the cavitation process, a considerable amount of energy release results in extremely high temperatures and strong shear stress in local areas, giving rise to shock waves, micro-jets, and other mechanical effects. At the same time, it causes changes in muscle tissue and protein structure, thereby affecting the quality and sensory characteristics of fish [6]. During cold storage, there is a significant reduction in the microbial population of salmon due to ultrasonic treatment. The same study noted that mechanical vibrations are generated by the ultrasound-altered microbial biofilm attached to aquatic organisms, preventing the microorganisms’ adhesion to product surfaces and limiting their increase [6].

An orthogonal ultrasonic field is the spatial sound field that results when the superimposition of two types of ultrasonic waves at 90°, emitted from opposing directions, occurs. Upon the application of orthogonal ultrasonic waves, particle vibrations in the medium will continue to be orthogonal to the sound wave’s propagation direction, rather than lessening each other, as described by the principle of acoustic wave superposition. In this case, improvements in the particle vibration intensity will take place [7,8]. Tian et al. [9] found that, compared with unidirectional ultrasound-assisted freezing (UAF), acceleration of the freezing speed materializes as a result of dual-frequency orthogonal UAF. The findings demonstrate that the cavitation effect of orthogonal ultrasound is larger than that of single ultrasound when the ultrasonic frequencies and powers are the same.

The majority of the existing literature on the ultrasonic processing of aquatic products has focused on horizontal dual-frequency ultrasound [10]. Sivakumar et al. [11] observed that, compared with single-frequency ultrasound, bubble growth was enhanced with a dual-frequency ultrasound at roughly the same overall intensity, meaning that it has higher levels of energy efficiency. According to X. Zhao, Lan et al. [12], LYCs can assist in slowing down the growth and reproduction of microorganisms and extend their storage period by using a 10 min dual-frequency ultrasonic treatment (175 W, 20/28 kHz). More specifically, on top of other valuable benefits, this treatment could curb microbial development, delay the increase in TVB−N and pH values, and maintain a larger water retention capacity throughout cold storage [13]. Amiri et al. [14] established that different ultrasonic powers (100 W and 300 W) and application times (10, 20, and 30 min) could significantly boost the emulsifying activity and stability of proteins. Of equal importance, Cheng et al. [15] noted that the UAF and potassium alginate (PA) treatments had several powers (160 W, 175 W, 190 W) that would affect LYCs in terms of the quality, including water retention capacity, quality characteristics, and myofibrillar protein (MP) performance. Weiqing Lan et al. [16] pretreated sea bass with US at 20 kHz and 600 W, and the outcomes confirmed that the combined treatment of US and SAEW yielded the best quality preservation compared with the control group. At present, ultrasonic refrigeration utilizing dual-frequency technology is widely applied. However, the frequency directions for its application are relatively simple, and there are only a few studies on power selection. Considering that there are a limited number of relevant studies, this paper attempted to identify the effects of orthogonal dual-frequency ultrasonic refrigeration with different powers on the quality of aquatic products.

To inhibit the metamorphism of LYCs, this research probed the impacts of orthogonal ultrasound with different powers. The quality of the fish was assessed by measuring the physicochemical properties of large yellow croaker fillets (LYCFs) during cold storage.

## 2. Materials and Methods

### 2.1. Preparation of Fillets from LYCs and Sample Processing

**Figure 1:**
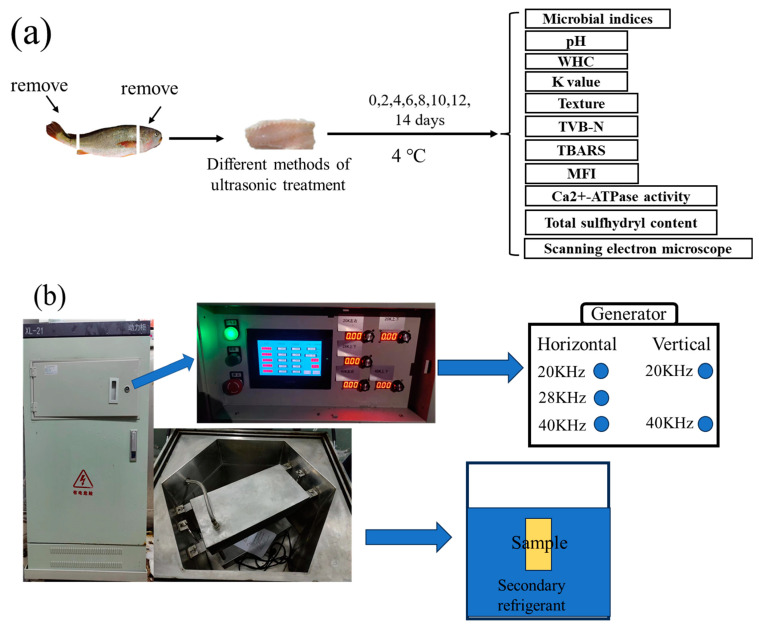
Experimental design diagram (**a**) and orthogonal ultrasonic assisted refrigeration system diagram (**b**).

This research obtained a total of 58 LYCs weighing an average of 500 ± 20 g in the same batch from the seafood market close to the authors’ university (Shanghai, China). Prior to being moved to the laboratory, oxygen-filled bags kept the LYCs alive. We immediately removed the head, guts, and skin upon stunning them with a strike on their heads (SHOU−DW−2023−302). Each fish was separated into two fillets of comparable size and shape, which were then washed with 1% NaCl solution. To determine the fundamental quality indices, the fillets were selected at random on Day 0. The batches of fillets were separated into groups handled under different powers; the (a) 200 W, (b) 300 W, and (c) 400 W treatment groups. We set the ultrasonic intermittent mode at 30 s on/30 s off cycle, ultrasonic treatment time at 10 min, and temperature of the secondary refrigerant (carrier refrigerant) at [29.3% CaCl_2_ brine solution (*w*/*v*)] 4 °C. A group soaked in secondary refrigerant deprived of any ultrasonic treatment was selected as the CK treatment group. Then, the samples were taken out, dried, packaged, stored at 4 ± 0.5 °C, and analyzed every two days. To exclude individual differences, we crushed and mixed four fillets from the same bag prior to measurement. Figure 1a shows the experimental design, as depicted by a conceptual diagram. Originally from Xiecheng Ultrasonic Equipment Co., Ltd. (Jining, China), the multi-frequency UAF instrument was designed by our team (Jining, China) (Figure 1b). The equipment processing groove size was a hexahedral inner groove, with an internal tangent circle diameter of 750 cm and depth of 600 cm; its capacity was 288 L, and common capacity was 200 L; the operating frequencies were 40 KHz, 28 KHz, and 20 KHz; and ultrasonic power was set to 400 W/group, with an adjustable power range of 40–100%.

### 2.2. Microbiological Analysis

To identify psychrophilic bacteria and gauge the total viable counts, we employed the approach of W. Liu et al. [17]. To dilute the sample, about 5 g was homogenized with 45 mL sterile saline. This was a tenfold serial dilution in view of the homogenous solution. Using a solution (100 μL), we diluted the plate count agar medium (PCA) that was used for culture. Subsequently, we recorded the number of psychrophilic bacteria and total viable counts after 2 days at 37 °C and one week at 4 °C in an incubator. This was carried out thrice, and each count was described by using Log CFU/g.

### 2.3. Determination of pH

To the 2 g of minced LYCFs, we added 18 mL of distilled water. Using a high-shear mixer, we then homogenized the mixture for 30 s (IKA homogenizer, Staufen, Germany). To test the pH level of the mixture, a pH meter (Mettler Toledo, Zurich, Switzerland) was utilized [18].

### 2.4. Determination of WHC

Approximately 2 g of the sample was wrapped in two layers of paper and placed in the centrifuge tube. For 10 min at 4 °C, we centrifuged them at 5000 rpm. Using filter paper, the water on the surface was dried. To obtain the WHC, the following equation was used [19]:

$$\mathrm{Water}\;\mathrm{holding}\;\mathrm{capacity}\;(\%)=\frac{meat\;mass\;after\;centrifugation}{meat\;mass\;before\;centrifugation}\times 100\%$$

### 2.5. TVB−N

TVB−N was measured with reference to P. Li, Peng et al. [20]. The TVB−N values were obtained using an automatic Kjeldahl nitrogen determination device (Kjeltec-8400, Foss, Hilleroed, Denmark).

### 2.6. Determination of TBA

The TBA was determined with reference to Zhou et al. [21]. We accurately weighed 5 g of fish samples, mechanically homogenized them, added 25 mL of 20% trichloroacetic acid. They were kept standing for 1 h, then centrifuged at 0 °C, 8000 r/min for 10 min, filtered, and the filtrate transferred to a 50 mL volumetric flask with a constant volume. We then used a pipette to transfer 5 mL of the above solution into a beaker. We added 5 mL of the 0.02 mol/L TBA reagent and mixed to combine. The mixture was reacted in a boiling water bath for 20 min, then removed and cooled to room temperature. Establishing distilled water as a control, we measured the absorbance value at 532 nm. Using the equation below, we calculated the TBA value:

TBA=0.78A

In the equation, TBA is expressed in terms of mg/kg, and A is the absorbance value at 532 nm.

### 2.7. K Value

Using a freshness detector (QS−3201, QS−SOLUTION, Tokyo, Japan), we identified the K value. To cut fillets to about 5 × 5 mm in size, we added 600 mL of extract A (extracting solution), and cut the fish with scissors (30 to 60 s). Then, to neutralize the sample’s pH, we added extract B (neutralizing fluid). In this case, we ensured that the pH was between 6 and 8 by using a pH test paper, and made sure the solution was neutral [22]. The supernatant was taken for electrophoretic operation after standing, and the K value was calculated.

### 2.8. Determination of Texture

Utilizing a texture analyzer (SMS TA. XT Plus, Stable Micro Systems, Ltd., Godalming, Surrey, UK), the adhesion, chewability, elasticity, and hardness of the fish were measured. The dimensions of the same part of the fish that was prepared into cubes were 20 × 20 × 15 mm. Each group was measured in triplicate. The measurement mode was TPA, the measuring probe was TPA P/36R cylindrical, the speed before and after the test was 1 mm/s, the test interval was 3 s, and the fish deformation rate was 30% [23].

### 2.9. Scanning Electron Microscope (SEM)

The samples of large yellow croaker were cut into 3 mm × 3 mm × 1.5 mm pieces with a Leica blade, then 2.5% glutaraldehyde solution was added and fixed at 4 °C for 24 h. The fixative solution was removed, and the samples were rinsed with 0.1 mol/L phosphate buffer with a pH value of 7.3 3 times for 15 min each time. Then, the samples underwent gradient elution with ethanol solution of 30%, 50%, 70%, 80%, 90%, 95%, and 100%, then the ethanol was replaced with isoamyl acetate and freeze-dried in a freeze-dryer (model FDU−2110, rated power AC220V, 50 Hz, 2.4 KVA, maximum working power 11A) for 3 days. Sections were observed under 5 kV scanning electron microscopy (SU5000, HITACHI, Japan).

### 2.10. Determination of Total Sulfhydryl Content

The total sulfhydryl content was defined by referring to the kit (Beijing Solaibao Technology Co., Ltd., Beijing, China), and the results were expressed as μmol/mg protein [24].

### 2.11. Determination of Ca^2+^−ATPase Activity

Ca^2+^−ATPase activity was assayed using the reference kit (Beijing Solaibao Technology Co., Ltd., Beijing, China). The outcome was expressed as U/mg protein.

### 2.12. Determination of Myofibril Fragmentation Index (MFI)

In reference to Culler et al. [25] and modified as appropriate, 2 g of fish and 20 mL of extract buffer were added, 12,000 r/min ice bath homogenization (30 s/time, twice) was performed, and 12,000 r/min refrigerated centrifugation for 15 min was carried out. We added 15 mL of MFI buffer into the precipitation after repeating the above process. Using double gauze, the subsequent step involved mixing and filtering. The myofibrillar protein solution was the filtrate. The protein concentration was adjusted to 0.5 mg/mL, and the absorbance at 540 nm was measured using the biuret method with an enzyme marker. The mean value was multiplied by 200 to obtain the MFI.

### 2.13. Statistical Analysis

This paper conducted three individual trials. After completing multiple comparisons, the means ± standard deviations were recorded, specifically by utilizing one-way ANOVA (analysis of variance) in SPSS 22.0.

## 3. Results and Discussion

### 3.1. Microbiological Analysis

**Figure 2:**
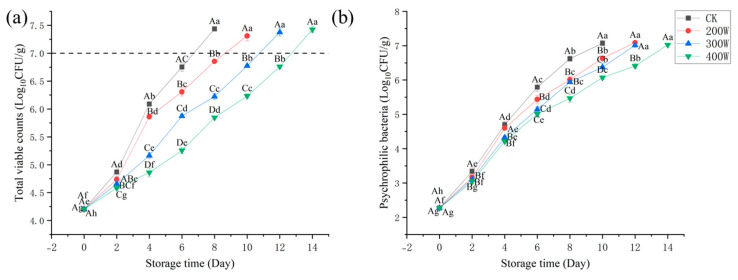
Changes in total viable count (**a**), psychrophile (**b**) of LYCs during refrigerated storage (4 °C). Different uppercase letters mean the same column for different days (*p* < 0.05); different lowercase letters mean the same column for different treatments (*p* < 0.05).

Figure 2 presents the results of microbiological analysis of the LYCFs. We measured the TVC on fresh large yellow croaker samples. On Day 0, with total viable counts of 4.20 lg CFU/g, fresh samples were observed (Figure 2a). Compared with the untreated samples, the total viable counts were lower in the ultrasonic samples despite the increase in total viable counts across all samples during storage [26,27]. In terms of aquatic product safety (7 logCFU/g, microbiological examination of food hygiene—detection of aerobic bacterial count), the total viable counts of CK exceeded the allowed level of microbiological spoilage on Day 8. Furthermore, on Day 10, the total viable counts of LYCs treated in 200 W reached 7.31 Log CFU/g. The total viable counts of LYCs in the 400 W treatment group were 6.28 Log CFU/g on Day 10, which exceeded the allowed level of microbiological spoilage. The 400 W treatment group exceeded the allowable level for fish of 7.43 Log CFU/g on the Day 14. Based on the findings, the growth of microorganisms can be effectively inhibited through ultrasonic treatment.

At 400 W ultrasound treatment, the optimal effect was observed. When stored in an aerobic environment at low temperature, the spoilage of fresh fish could mainly be attributed to the bacteria known as psychrophilic bacteria. On Day 0, its count was 2.27 Log CFU/g in the CK treatment group, which gradually increased throughout the storage phase (Figure 2b). In relation to those of CK and other treatment groups, the increase in these specific bacteria slowed significantly in the 400 W treatment group. On Day 10, the psychrophilic bacteria in the CK treatment group reached 7.07 Log CFU/g, while the three treatment groups (200 W, 300 W, and 400 W) were 6.63, 6.37, and 6.07 Log CFU/g, respectively.

Generally, ultrasonic pretreatment slowed the growth of microorganisms on stored LYCFs, and increasing ultrasonic power enhanced the effect of inhibiting bacterial growth. This result underlines that the orthogonal double-frequency 400 W treatment group has the most optimal effect on delaying the growth of total viable counts and psychrophilic bacteria.

### 3.2. pH Value

**Figure 3:**
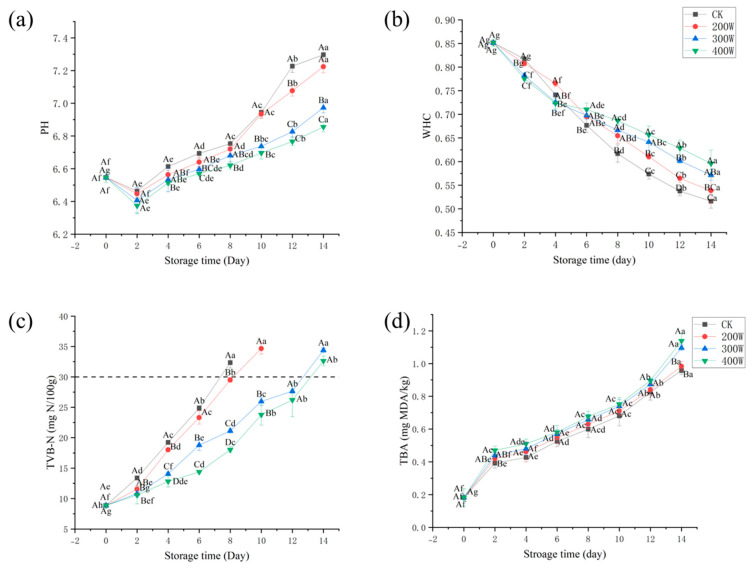
Changes in pH value (**a**), WHC (**b**), TVB−N (**c**), and TBA (**d**) of LYCs during cold storage. Different uppercase letters mean the same column for different days (*p* < 0.05); different lowercase letters mean the same column for different treatments (*p* < 0.05).

When assessing the freshness of aquatic products, we can use pH values, since they are influenced by enzymatic and microbial activities. Over the first 48 h of storage, a decrease in the pH value of LYCFs was noted. However, it increased throughout the remaining storage time (Figure 3a). The buildup of lactic acid and release of inorganic phosphate led to a lower pH value. Subsequently, degradation arising from microorganism activity was associated with a higher pH value [28,29]. The pH value will affect the activity of enzymes and the state of the microbial cell membrane with charge, change the permeability of the cell membrane, and affect the absorption of nutrients and the excretion of metabolites by microorganisms. Therefore, different pH values often cause different metabolic processes in bacteria, so that the quality and proportion of metabolites are changed. The creation of alkaline compounds like other alkaline nitrogen, trimethylamine, and ammonia is caused by the use of amino acids during microbial development, thereby increasing the pH level. Due to the bactericidal properties of ultrasound, ultrasound-treated samples consistently maintained a lower pH value than that of the CK treatment group [30].

### 3.3. WHC

Throughout the storage of the fillets, their WHC significantly decreased (Figure 3b). The underlying cause behind such a decline could be the unpredictability of fish protein–water interactions, bacterial growth, degradation of MP, and changes in muscle structure. Given these conditions, a decrease in WHC and an increase in extracellular space were observed [31]. The WHC of fresh LYCs was 85.14 ± 0.74%. Moreover, the WHC of the CK treatment group and 200 W treatment group decreased exponentially during storage and was lower than those of the other two treatment groups by Day 6. Bacteria in fish may reproduce late in storage, causing rapid protein breakdown and water loss. Among them, the WHC of the CK treatment group decreased to 51.63 ± 1.48% on Day 14, while that of the 200 W, 300 W, and 400 W treatment groups remained at 53.86 ± 2.71%, 57.13 ± 1.15%, and 59.58 ± 0.03%, respectively. At the end of the storage period, the WHC of the 400 W treatment group was significantly higher than those of the other treatment groups.

### 3.4. TVB−N

With the majority of its components being TMA, nitrogen from ammonia, and dimethylamine, TVB−N is linked to endogenous microorganisms and enzymes [32]. It is commonly used to examine the freshness of aquatic products [33]. Numerous investigations have discovered that TVB−N concentration is significantly correlated with the decrease in freshness due to microbes [34,35]. As displayed in Figure 3c, 8.87 mg N/100 g was the TVB−N value of the fresh LYC sample. For the CK treatment group on Day 8, the TVB−N value increased to 32.34 mg N/100 g, which exceeded the acceptable limit of 30 mg N/100 g (Commission Implementing Regulation (EU) 2019/627). Nevertheless, the values for such croakers were lower than the allowable value, despite being treated with 300 or 400 W ultrasound on Day 12. According to the results, during chilled storage, TVB−N was significantly decreased by the 400 W treatment group, which could be linked to the antibacterial property of the ultrasound.

### 3.5. TBA

For the LYCs, Figure 3d presents their TBA value when stored at 4 °C, which specifies the extent to which the lipids were oxidized. Regarding unsaturated fatty acid oxidation, one of its breakdown products is MDA (malondialdehyde). The degree of oxidative rancidity of aquatic lipids is shown by persistent red molecules, which could then be created through the interaction of TBA with MDA [36].

Figure 3d underscores that all treatment groups showed an increasing trend during refrigeration, which was primarily affected by the degree of deterioration of the LYCFs [37]. Significant differences were found between groups at the beginning (on Day 4) and end (on Day 14) of storage. The extension of cold storage time made the deterioration of samples prevalent, accelerated the oxidation of fatty acids, and increased the TBA value. In the early storage period, TBA values were higher in all treatment groups than in the CK treatment group. Take Day 8 of mid-storage for example, the TBA of the 200 W treatment group was 0.63 ± 0.03 mg MDA/kg, while that of the 300 W treatment group was 0.66 ± 0.04 mg MDA/kg. Additionally, it was 0.68 ± 0.03 mg MDA/kg in the 400 W treatment group and 0.60 ± 0.05 mg MDA/kg in the CK treatment group. These findings may stem from the active oxygen generated by ultrasound, which can trigger the oxidation of lipid free radicals, thereby increasing of MDA content. Moreover, lipid oxidation becomes increasingly intense with the increase in ultrasonic power [32].

### 3.6. Texture

**Figure 4:**
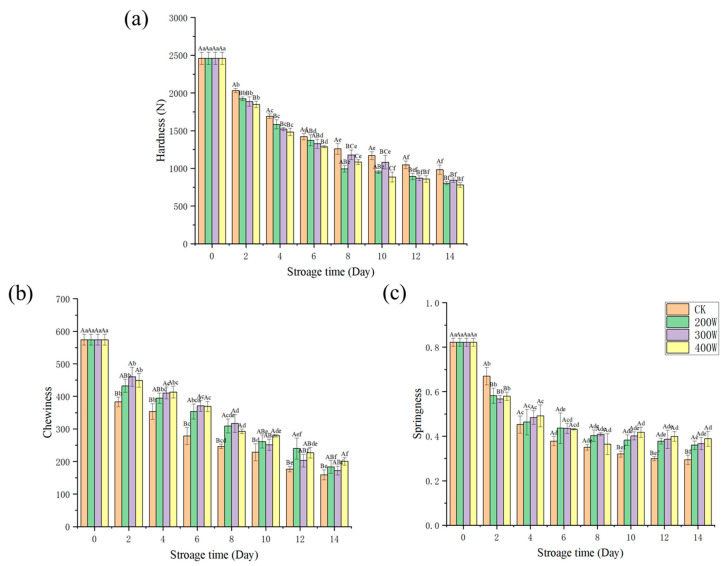
Changes in hardness (**a**), chewiness (**b**), and springiness (**c**) of LYCs during cold storage. Different uppercase letters mean the same column for different days (*p* < 0.05); different lowercase letters mean the same column for different treatments (*p* < 0.05).

As a result of the degraded and denatured protein of the fish samples during storage, particularly to varying degrees under the actions of microorganisms, endogenous enzymes, and trypsin, we witnessed a decrease in the elasticity, chewability, and hardness values of the fish in every treatment group, which occurred with the increase in storage duration. It should be pointed out that the texture properties underwent a significant reduction, and the muscle connective tissues and fibrin were destroyed [38]. With regard to the samples, their initial chewability, elasticity, and hardness values were 574.37 ± 16.76, 82 ± 0.02, and 2459.81 ± 81.37 N, respectively. Compared with that of the CK treatment group, the 300 and 400 W treatment groups had hardness values that significantly differed throughout the entire storage period. Consistent with the shift in MFI value, ultrasound had substantial effects on the hardness of fish and could alter its tenderness. This arose from the hardness of the ultrasonic groups being significantly lower than that of the CK treatment group. Inside the muscle, the impulse and pressure were dramatically increased by the hole effect of ultrasonic waves. To some extent, it also disrupts the integrity and spatial structure of proteins, which reduces the performance of muscle proteins [39,40]. The results were significantly different (Figure 4, *p* < 0.05).

### 3.7. Scanning Electron Microscope

**Figure 5:**
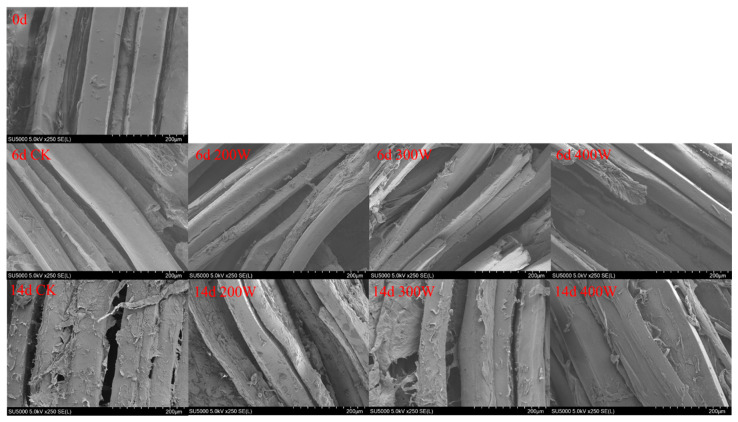
Scanning electron microscope (at 250×) of LYC strips under different ultrasound powers.

The microstructure analysis of LYC samples post-ultrasonic treatment evidenced that the muscle changed with the increase in ultrasonic power (Figure 5). The arrangement of muscle fibers became disordered, the distance between muscle bundles widened, the muscle structure became relaxed, and even the phenomenon of cell rupture took place. Ultrasonic cavitation and the mechanical effect caused this phenomenon. Figure 5 shows a surface electron microscope image of the sample, magnified 250 times. Additionally, gaps began to appear in myofibers of all groups at Day 6, with the gaps at 200 W and 300 W being the largest. When the power was 300 W, the rupture degree of muscle fiber was the largest, but when the ultrasonic power was 200 W and 400 W, it was lower, which may be due to the ultrasound within this power range limiting the damage degree of LYC muscle fiber.

### 3.8. K Value

**Figure 6:**
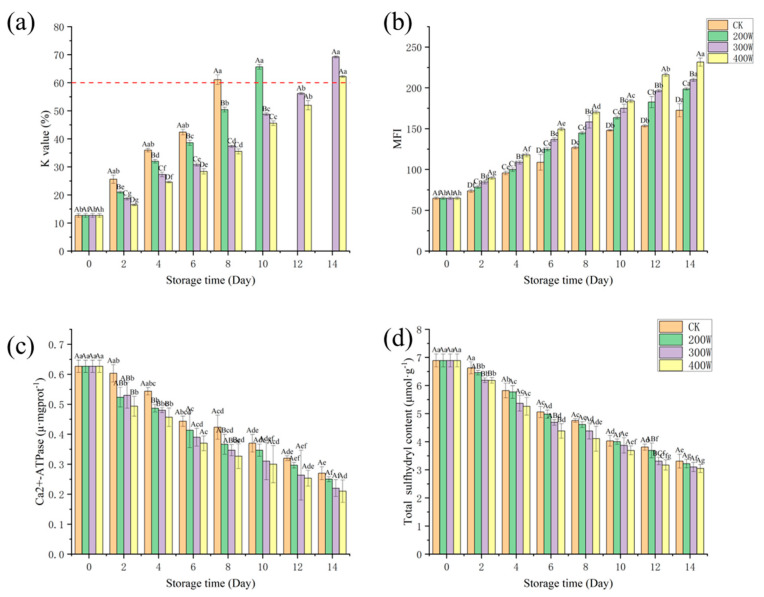
Influence of ultrasound power on the K value (**a**), MFI (**b**), Ca^2+^−ATPase (**c**), and total sulfhydryl content (**d**) of LYCs during cold storage. Different uppercase letters mean the same column for different days (*p* < 0.05); different lowercase letters mean the same column for different treatments (*p* < 0.05).

The K value is utilized to evaluate the freshness of fish. When higher than 60%, the fish is deemed completely rotten [41]. The K value of the fresh LYC fillet was 12.67% (Figure 6a), and the results were significantly different between groups (*p* < 0.05). It increased because of the decomposition of ATPase and its derivatives [20]. The K value of the CK treatment group exceeded the upper limit on Day 8, while the 200 W treatment group surpassed the upper limit on Day 10. At the end of the storage, the K value of the 300 W and 400 W samples exceeded the upper limit, and the K values of the 400 W samples were consistently lower than those of the other three treatment groups during storage [42]. As storage time increases, ATP is degraded to HxR and Hx, which ultimately leads to the production of spoilage substances. This ultimately leads to the development of a spoilage flavor in fish. With the increase in ultrasonic power, the effect of inhibiting microbial growth to delay the degradation of inosinemonphosphate (IMP) and the production of HxR and Hx improved, causing the low K value in 400 W treatment group samples.

### 3.9. MFI

MFI is an indicator of the integrity of the myofibrillar protein, and a larger MFI value may be associated with the severe rupture of myofibrillar fibers near the Z-line of the I zone. Equally essential is MFI, which is a vital indicator characterizing meat tenderness, and its value is proportional to the degree of myofibrillar fracture and meat tenderness [43]. As shown in Figure 6b, the MFI values of all samples increased with the extension of storage time, and the MFI values of the CK treatment group and other treatment groups were significantly different, which is consistent with the conclusion of Hopkins et al. [44]. This may have emerged due to the activation of calpsin in the muscle after the fish dies, causing the degradation of Z-disk-related myofibrillar protein. Consequently, it results in muscle fiber breakage [45].

The MFI value was positively correlated with ultrasonic power. For the 400 W treatment group, the MFI value was significantly higher than those of the other treatment groups. It could be that the higher the ultrasonic power, the more potent the cavitation effect of the ultrasound, resulting in the (a) extensive destruction of the myofibril and connective tissue of the fillet, (b) accelerated fragmentation of the myofibril, and (c) corresponding increase in the MFI value [46]. With the increase in power, the MFI of the LYCFs significantly increased, implying that the effect of ultrasonic treatment on the MFI of the LYCFs was related to its power, and the increase in ultrasonic power could drastically improve the tenderness of LYCFs. This was closely related to cavitation and mechanical effects, which can produce a strong shear effect and severely damage the myofibrillar protein in a relatively short time. As a result, it induces structural weakening and fracture, thereby improving meat tenderness.

### 3.10. Ca^2+^−ATPase

Myosin integrity can be reviewed by measuring Ca^2+^−ATPase activity, as a decrease in protein mass leads to an increase in enzyme activity. Figure 6c indicates that the Ca^2+^−ATPase values of samples in each treatment group presented a downward trend with the increase in storage time. The results were significantly different between groups (*p* < 0.05). The oxidation of the mercapto treatment group of the globular head of myosin and the aggregation of myosin may have caused this trend. With the increase in storage time, the Ca^2+^−ATPase value in the ultrasound treatment group decreased at a faster rate than in the control and treatment groups. On Day 14 of storage, Ca^2+^−ATPase values in the 200 W, 300 W, and 400 W treatment groups sharply decreased markedly to 0.25 ± 0.01, 0.22 ± 0.03, and 0.21 ± 0.04 U/mg, respectively, while 0.27 ± 0.02 U/mg was the value for the CK treatment group.

### 3.11. Total Sulfhydryl Content

In proteins, one of the leading functional treatment groups is sulfhydryl. A crucial indicator of protein oxidation in muscles is the change in its content [47]. Across all sample treatment groups, we witnessed a downward trend in total sulfhydryl content (Figure 6d), which could be construed as an indication of the growth in disulfide bonds. The content was significantly different between groups (Figure 6d, *p* < 0.05). On Day 14, it fell to 3.31 ± 0.25 μ mol/g in the CK treatment group, 3.22 ± 0.15 μ mol/g in the 200 W treatment group, 3.10 ± 0.17 μ mol/g in the 300 W treatment group, and 3.05 ± 0.14 μ mol/g in the 400 W treatment group. The loss of total sulfhydryl content was found to be the largest in the 400 W treatment group since ultrasonic treatment can extinguish the protein structure of the sample. With the increase in ultrasonic power, protein molecules gradually expand. As a result, the internal sulfhydryl treatment group becomes exposed, which then promotes the synthesis of disulfide bonds, causing a rapid decrease in the total sulfhydryl content [48].

## 4. Conclusions

In this paper, the effects of orthogonal ultrasonic pretreatment under different powers on the total viable counts, TVB−N value, TBA value, pH value, and K value of LYCFs during refrigeration at 4 °C were investigated. The results proved that the use of orthogonal ultrasound therapy can significantly reduce the development of bacteria. Additionally, when the ultrasonic power was increased, the antibacterial effect progressed over time. The storage times at which total viable counts increased in LYCs in the CK, 200 W, 300 W and 400 W groups were 8 days, 10 days, 12 days, and 14 days, respectively. TVB−N and K values increased at the storage times of LYCs in the CK, 200 W, 300 W and 400 W groups of 8, 10, 14, and 14 days, respectively. Decreasing total sulfhydryl content and Ca^2+^−ATPase activity, as well as facilitating the formation of disulfide bonds, expanded the protein and slightly damaged the sample’s protein structure due to the increase in ultrasonic power. Compared with other groups, the 400 W group proved the most effective method to inhibit the deterioration of LYCs. Therefore, it is recommended to use the 400 W pretreatment method based on orthogonal dual-frequency to extend the shelf life of LYCs.

## Data Availability

The data presented in this study are available on request from the corresponding author.
